# Direct and indirect effects of hepatitis B vaccination in four low- and middle-income countries

**DOI:** 10.1016/j.epidem.2024.100798

**Published:** 2024-12

**Authors:** Margaret J. de Villiers, Edward de Villiers, Shevanthi Nayagam, Timothy B. Hallett

**Affiliations:** a MRC Centre for Global Infectious Disease Analysis, School of Public Health, Imperial College London, London, United Kingdom; b Teddington, Middlesex, United Kingdom; c Division of Digestive Diseases, Department of Metabolism, Digestion and Reproduction, Imperial College London, London, United Kingdom

**Keywords:** Vaccine impact, Indirect effect, Herd effect, Hepatitis B vaccination, Hepatitis B birth dose, Infectious disease modelling

## Abstract

Population-level vaccination effects of the hepatitis B vaccine were investigated in four low- and middle-income countries with different levels of vertical and horizontal transmission. Indirect vaccination effects constitute a large proportion of overall vaccination effects of the vaccination programmes in all four countries (over 70% by 2030 in all four countries). However, countries with higher levels of vertical transmission benefit less from indirect vaccination effects from the infant hepatitis B vaccine series during the first decades of the vaccination programme, making the birth dose vaccine more important in these countries. Vaccination, even at levels that do not fully control transmission, has a great effect on the development of disease as it also increases the average age of infection, thereby causing a decrease in the number of chronic infections relative to the number of acute infections.

## Highlights

• The ratio of direct to indirect effects depends on population characteristics.
• Nevertheless, indirect effects will dominate within a few years.
• When vertical transmission is significant, birth dose is especially effective.
• Birth dose is, moreover, vital in reducing chronic infections.

## 1 Introduction

Vaccination against vaccine-preventable diseases is estimated to save about 97 million disability adjusted life years (DALYs) per year and six million lives per year, having the potential to save a further 3 million lives per year if vaccination coverage were increased globally (Ehreth, 2003). It is estimated that vaccination for ten vaccine-preventable infectious diseases saved 50 million lives between 2000 and 2019 in 112 low- and middle-income countries (LMICs) (Toor et al., 2021). By affording individuals long-term protection from an infectious disease, a vaccine not only reduces morbidity and mortality from the infectious disease, but also reduces the long-term sequelae associated with infection, along with the accompanying medical interventions and downstream costs.

Hepatitis B is a long-lasting viral infection of the liver that can cause liver cirrhosis and cancer. The incubation period is 30 to 180 days. The initial stages of infection tend to be characterised by high levels of viral replication, with both HBsAg (hepatitis B surface antigen) and HBeAg (hepatitis B e-antigen) detectable in the blood, and high levels of infectiousness. Subsequent steep declines in viral replication result in the clearance of HBeAg, and patients that are HBeAg negative tend to be less infectious. Infection can be transmitted vertically from an infected mother to her child at birth or horizontally from person to person via contaminated blood or bodily fluids (World Health Organization, 2017). The development of chronicity (HBsAg detectable in the blood for more than 6 months) is correlated strongly with age, with infections far more likely to become chronic in infants and young children than in older children and adults (Edmunds et al., 1993). Hepatitis B infection induces an immune response that causes liver damage, with chronic sufferers having an elevated risk of premature mortality due to liver cirrhosis or hepatocellular carcinoma. The World Health Organisation (World Health Organization, 2024b) estimates that there were 254 million chronic sufferers and 1.1 million HBV-related deaths globally in 2022. In 2022, HBsAg prevalence in the six WHO geographical regions ranged from 0.5% in the Americas (PAHO) to 5.8% in the African region (AFRO) (World Health Organization, 2024a).

Vaccination for hepatitis B was introduced in 1981 (Tulchinsky, 2018) and is highly effective (Ni et al., 2001, Liang et al., 2009, Chen et al., 2012, Peto et al., 2014). Timely birth dose (HepB-BD; administered within 24 h of birth) provides short-term protection against vertical infection from infected mothers to their infants. However, three or four doses of hepatitis B vaccination (HepB3) are needed for lasting immunity (at least 20 or 30 years) from horizontal infection (World Health Organization, 2019). Hepatitis B vaccination programmes are estimated to have saved 29 million lives between 2000 and 2030 in 112 LMICs (Toor et al., 2021), and are expected to have a much greater impact in the future due to the large lag between infection and death. de Villiers et al. (2021) found that scaling up HepB-BD coverages to 90% by 2030 in 110 LMICs while maintaining the HepB3 coverages at their 2019 values could avert about 41 million chronic infections between 2020 and 2100 and save approximately 710 thousand lives in the 2020 to 2030 birth cohorts compared to maintaining both HepB3 and HepB-BD coverage levels at their 2019 values.

In 2016, the WHO committed to eliminating hepatitis B as a public health threat by 2030. To achieve this, it set out five service coverage targets, including increasing both HepB-BD and HepB3 vaccination coverage by 2030 to 90% of all of those eligible (World Health Organization, 2016). While HepB3 coverage is high in most countries — globally 84% in 2022, ranging from 72% in AFRO to 93% in the WHO Western Pacific region (WPRO) — HepB-BD coverage is far more limited and patchy — globally 45% in 2022 (World Health Organization, 2023), ranging from 18% in AFRO to 80% in WPRO (World Health Organization, 2024a). Since high HepB-BD coverage is important for accelerating the elimination of hepatitis-B — de Villiers et al. (2021) found that hepatitis B elimination could be brought forward by about 50 years in both AFRO and the WHO’s Middle East region (EMRO) by scaling up HepB-BD coverage to 90% by 2030 in LMICs under 2019’s status quo HepB3 coverage levels — scaling up HepB-BD coverage in LMICs over the next few years is essential.

During vaccine development and subsequent public health planning and cost-effectiveness analysis, it is necessary to quantify vaccine effectiveness against an infectious disease. A vaccine can protect individuals in the population against infection in different ways (Halloran et al., 2010). *Direct* vaccine protection refers to the protection from infection afforded by the vaccine to a vaccinated individual during subsequent encounters with infectious individuals. However, the greater the proportion of the population that is immune to infection due to successful vaccination or to recovery from infection, the less disease transmission will occur in the population, resulting in a fall in disease prevalence. Individuals (both vaccinated and unvaccinated) are therefore less likely to encounter an infected individual, resulting in the second form of protection from vaccines — *indirect* protection. *Overall* vaccine protection is protection (both direct and indirect) from infection provided by the vaccine to the whole target population, which contains both vaccinated and unvaccinated individuals. In contrast, *total* vaccine protection only considers both direct and indirect protection from infection provided by the vaccine to the vaccinated members in the target population. Hence, total protection (per person for the vaccinated group in the target population) will always be greater than overall protection (per person for everyone in the target population), with total and overall protection only being equal if everybody in the target population has been successfully vaccinated.

In field experiments to determine vaccine effectiveness, participants are partitioned between a control population in which no vaccination occurs and a target population which receives the vaccine (in practice, the control and target populations are often the same population pre- and post- the introduction of vaccination in the case of diseases with short latent and infectious periods). Vaccination effects are then calculated by finding the difference in scaled disease impact (where scaled disease impact is number of new infections in a group scaled by group size) between: the control and target populations (overall vaccination effects), the unvaccinated and vaccinated groups in the target population (direct vaccination effects), the control population and the unvaccinated group in the target population (indirect vaccination effects e.g. Khanam et al., 2023), and the control population and the vaccinated group in the target population (total vaccination effect).

More recently, dynamic compartmental disease transmission models are increasingly being used to disentangle vaccination effects of infectious diseases by comparing disease impact in the successfully vaccinated group versus the group unprotected by vaccination (unvaccinated and those for whom vaccination does not afford any benefit) in the modelled population. Eichner et al. (2017) calculated the direct effects in the case of influenza vaccination in Germany as all infections arising in successfully vaccinated individuals when the vaccine is set to be ineffective versus when the vaccine is set to be effective. A drawback of this approach is that effective vaccination also reduces disease prevalence, resulting in some successfully vaccinated individuals never encountering infectious individuals. This approach therefore ignores the fact that both direct and indirect effects protect vaccinees from contracting infection, thereby potentially over-estimating direct effects of the vaccine.

Brisson and Edmunds (2003) estimated indirect vaccine effects by comparing static model results with dynamic model results. Static models apply a constant force of infection (FOI) and therefore only model direct vaccine effects, whereas the FOI in dynamic models is dampened down by decreasing disease prevalence, which results in both direct and indirect vaccine effects being modelled. Hence, the difference in number of infections between static and dynamic models can provide an estimate for indirect effects. Similarly, vaccine effects have been investigated using dynamic compartmental disease transmission models by retaining direct effects but de-activating indirect vaccine effects. More specifically, Dhankhar et al. (2015) and Arinaminpathy et al. (2017) each used the FOI from the simulation containing no vaccination in the simulation containing vaccination in order to compare the number of infections under vaccination in the absence of indirect effects in their respective diseases. However, such an approach may lead to the indirect effects that benefit those vaccinated to be attributed to a direct effect. In this current study we investigate vaccine effects in hepatitis B by de-activating direct effects and retaining indirect effects.

The aim of this study was to investigate hepatitis B vaccination effects in four LMICs selected for having different demographic and epidemiological characteristics in terms of vertical and horizontal transmission. The effect of vaccination on the relative prominence of acute and chronic infections was also investigated in these countries.

## 2 Materials and methods

Vaccination effects of the HepB3 and HepB-BD vaccines were investigated using the age-structured population-level deterministic dynamic compartmental hepatitis B model described in Nayagam et al. (2016) and de Villiers et al. (2021) in MATLAB (The MathWorks Inc., 2023). The model was populated with country-specific demographic data (fertility rates, male-to-female sex ratios at birth, population size, migration rates, and all-cause mortality rates) between the years 1950 and 2100 from the United Nations World Population Prospects (2019) (United Nations, Department of Economic and Social Affairs, Population Division, 2019). Country-specific historical vaccination coverage (1980 to 2021) of HepB3 and timely HepB-BD vaccines were sourced from WHO/UNICEF (World Health Organization (WHO)/United Nations Children’s Fund (UNICEF), 2021). The country-specific prevalences (HBsAg and HBeAg/HBsAg) and death rates (deaths from cirrhosis and hepatocellular carcinoma) data used in model-fitting and the model-fitting algorithm used are described in de Villiers et al. (2021). For each country, 100 particles were sampled from the posterior distribution from the model-fitting, and results from these 100 particles were summarised as the mean as well as the 2.5% and 97.5% percentiles to form 95% credible intervals.

The number of cumulative incident infections (I) within each birth cohort was calculated in three different simulations:

• vaccination coverage was set to 0% so that there was no vaccination protection ($I_{\text{NoVacc}}$);
• vaccination coverage was enabled and all successfully vaccinated vaccine recipients were protected from infection ($I_{\text{Vacc}}$); and
• vaccination coverage was enabled but the effect of vaccination was modified such that successfully vaccinated vaccine recipients could still become infected but would not become infectious, thereby maintaining indirect vaccine protection but disabling direct vaccine protection ($I_{\text{Mod}}$).

Since direct and indirect vaccination effects are both disabled in $I_{\text{NoVacc}}$ and both enabled in $I_{\text{Vacc}}$, the difference will yield the overall vaccination effect in the population ($E_{\text{overall}}$). Since only indirect vaccination effect is enabled in $I_{\text{Mod}}$, whereas both direct and indirect vaccination effects are enabled in $I_{\text{Vacc}}$, the difference will yield the direct vaccination effect in the population ($E_{\text{direct}}$). The difference between $E_{\text{overall}}$ and $E_{\text{direct}}$ gives the indirect vaccination effect ($E_{\text{indirect}}$).

Vaccination effects for cumulative incident infections in a birth cohort standardised by the size of the birth cohort, $N_{\text{birth cohort}}$, were therefore calculated as follows:

(1) $$E_{\text{overall}}=\frac{I_{\text{NoVacc}}-I_{\text{Vacc}}}{N_{\text{birth cohort}}}\times 100\text{\%}$$

(2) $$E_{\text{direct}}=\frac{I_{\text{Mod}}-I_{\text{Vacc}}}{N_{\text{birth cohort}}}\times 100\text{\%}$$

(3) $$E_{\text{indirect}}=\frac{I_{\text{NoVacc}}-I_{\text{Mod}}}{N_{\text{birth cohort}}}\times 100\text{\%}=E_{\text{overall}}-E_{\text{direct}}$$

Hence, $E_{\text{overall}}$ measures the protection in a target population compared to in a control population, $E_{\text{direct}}$ measures the percentage of the birth cohort that is successfully vaccinated that would have been infected by subsequent encounters with infectious individuals but for them having been vaccinated, and $E_{\text{indirect}}$ measures the percentage of the birth cohort (vaccinated or unvaccinated) that does not get infected due to the vaccination programme reducing disease prevalence. Similar calculations were done in the case of cumulative chronic infections within birth cohorts.

**Fig. 1:**
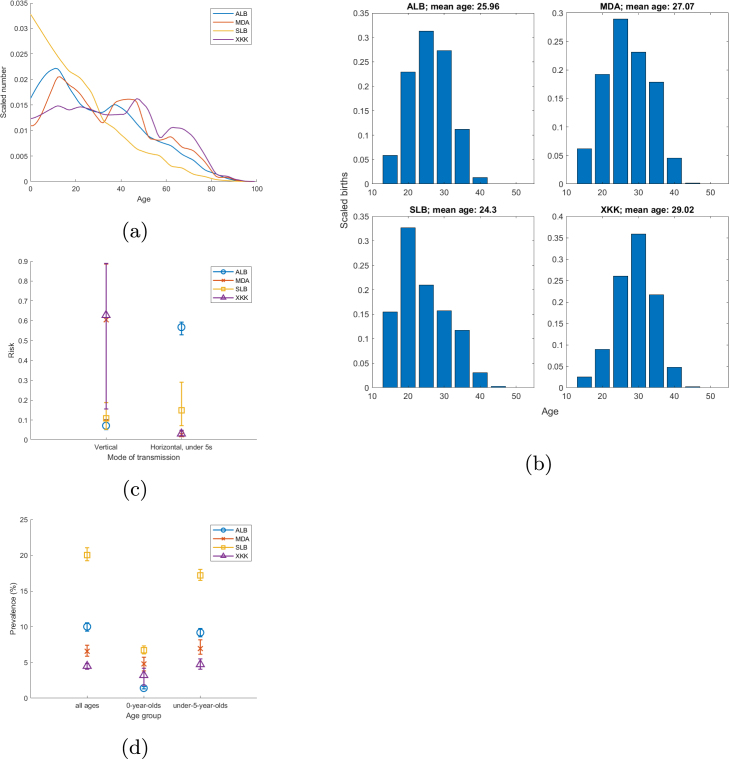
Demographic and epidemiological indicators. (a) Age profiles of the population (scaled by the total population) in the year 2000, when HepB3 vaccination begins in the stylised scenarios. (b) Number of births by age of the mother (scaled by the total number of births) in the year 2030. The mean age of a mother of a newborn is given for each country in the plot’s title. (c) The model-estimated transmission risk (with 95% credible intervals) from an HBsAg+ HBeAg- mother to her newborn under no intervention (*Vertical*) and the annual risk of an infectious under-five-year-old being horizontally infected under no intervention (*Horizontal, under 5s*). (d) HBsAg prevalence in different age groups in the year 2000 under no intervention. ALB: Albania; MDA: Moldova; SLB: Soloman Islands; XKK: Kosovo.

Vaccination in hepatitis B is complicated in that the timing and the number of vaccine doses determines protection from different transmission routes (vertical and horizontal), each of which is likely to result in different levels of direct and indirect protection from infection. Newborns that successfully received HepB-BD are protected from vertical infection but are subsequently vulnerable to horizontal infection, and uninfected six-month-old infants that successfully received HepB3 have lifelong protection from horizontal infection. This study measures the impact of the combined hepatitis B vaccination (HepB-BD and HepB3); the effects of each vaccine is not studied in isolation.

The simulations were run from the year 1890 to 2150 and vaccination effects were calculated for the birth cohorts born from 1980 to 2070. Because hepatitis B infection can last for decades, the simulations were initiated in 1890 to allow the simulations to reach equilibrium before vaccination started after 1980. To enable the simulations to start in the year 1890 and run until 2150, the demographic data for the year 1950 were applied to each of the years 1890 to 1949 and the demographic data for the year 2100 were applied to each of the years 2101 to 2150 in the simulations.

Four LMICs with similar population sizes were chosen to represent a range of demographic and epidemiological characteristics (Fig. 1): Albania (ALB), Moldova (MDA), Solomon Islands (SLB) and Kosovo (XKK).

**Fig. 2:**
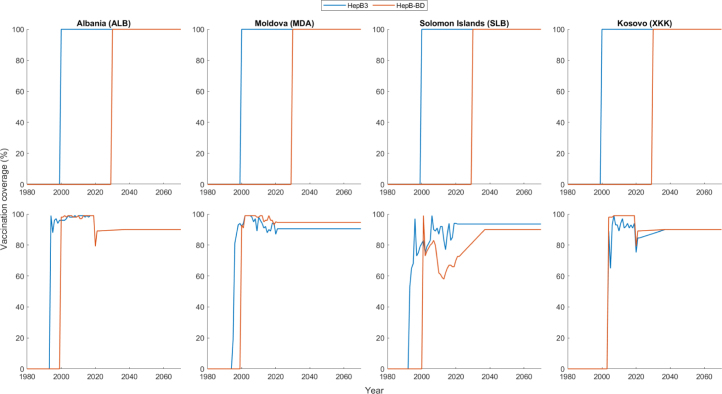
Vaccination scenarios. Stylised (top row) and aspirational (bottom row) vaccination scenarios.

Two vaccination scenarios were used (Fig. 2): a stylised scenario and a more realistic scenario. In order to first gain an understanding of the different types of vaccination impact, we constructed a *stylised* scenario whereby vaccination coverage was kept at 0%, before HepB3 vaccination coverage was increased from 0% to 100% in the year 2000 and HepB-BD coverage was increased from 0% to 100% in the year 2030. HepB3 and HepB-BD coverage were each kept at 100% until 2150, resulting in almost all vaccinated individuals being successfully vaccinated (in the model, the vaccination of an individual is either successful, in which case he is fully protected, or it fails, in which case the infection risk to the person is the same as that for an unvaccinated person (Smith et al., 1984)). Waiting 30 years before introducing HepB-BD enables the assessment of the incremental impact of HepB-BD in the presence of well-established HepB3. We then ran the analyses using the *aspirational* HepB3 and HepB-BD coverages: the historical HepB3 and HepB-BD coverages for each country up until the year 2021, after which HepB3 and HepB-BD were each maintained at the last recorded coverage value in 2021 if this value is greater than or equal to 90% (the WHO goal for vaccination coverage by the year 2030; World Health Organization (2016)), or HepB3 and HepB-BD coverage were each linearly scaled up to 90% over 15 years if the last recorded coverage value in 2021 is less than 90%.

**Fig. 3:**
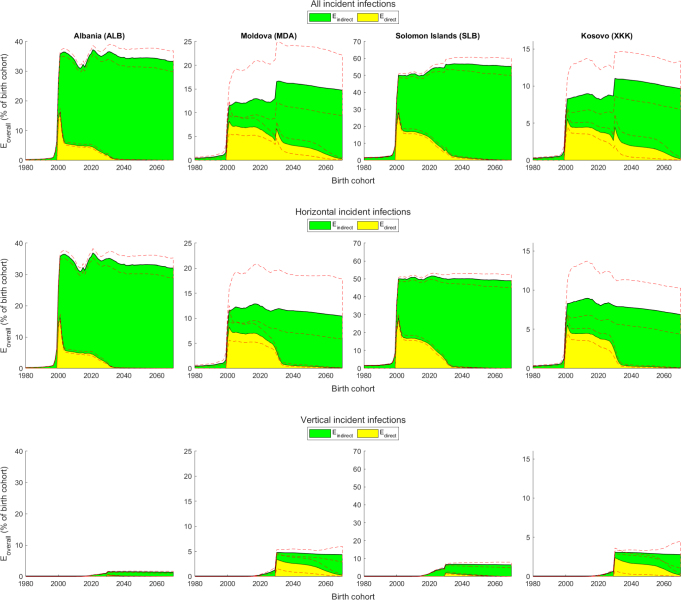
Vaccination effects ($E_{\text{overall}}$, $E_{\text{direct}}$ and $E_{\text{indirect}}$) amongst all infections (acute and chronic) under the stylised vaccination scenarios. Vaccination effects (with 95% credible intervals indicated by red dashed lines) as a percentage of birth cohort size in all (top row), horizontal (middle row) and vertical (bottom row) incident infections under the stylised vaccination scenarios. (For interpretation of the references to colour in this figure legend, the reader is referred to the web version of this article.)

## 3 Results

### 3.1 Vaccination impact in the stylised scenario

Overall vaccination effects for incident infections in the stylised vaccination scenarios (top row in Figs. 3 and A.3) respond immediately to the introduction of HepB3 vaccination in the year 2000, subsequently fluctuating as vaccinated birth cohorts age and immunity reaches older age groups. By the time HepB-BD vaccination is introduced in the year 2030, HepB3 has been providing both direct and indirect protection to the population for 30 years, reducing the proportion of pregnant women that is infected and therefore reducing vertical infection in the population. HepB-BD vaccination has the most noticeable effect on overall vaccination effects for incident infections in the countries with the highest risks of vertical transmission (Moldova and Kosovo; Fig. 1(c)). Note that the birth cohorts born before the initiation of the vaccination programme (prior to the year 2000) also benefit to some degree from the overall vaccination effects due to vaccinated individuals in later birth cohorts providing indirect protection to unvaccinated individuals in the earlier birth cohorts.

The percentage vaccination effect for incident infections that is direct (yellow portions in the top rows in Figs. 3, A.1 and A.3) is high initially (birth cohorts 2000–2003) because the first few birth cohorts exposed to vaccination are young and vulnerable when immunity due to vaccination is being introduced into the population, necessitating direct protection from vaccination to avoid infection. (At the same time, indirect effects also benefits the first few vaccinated birth cohorts, since some individuals are exposed to infection for the first time later in life, when immunity due to vaccination is more widespread in the population.) The percentage of direct vaccination effects for incident infections falls steeply over the first few birth cohorts after the introduction of HepB3 because of the increasing percentage of under-five-year-olds, a group that experiences the highest horizontal transmission rates, that acquire protection from HepB3 vaccination, resulting in falling disease prevalence in this age group and so growing indirect effects. This steep decline in the direct effect of vaccination is most noticeable in the countries with the highest risks of horizontal transmission amongst under-five-year-olds (Albania and Soloman Islands; Fig. 1(c)). The percentage of direct vaccination effects for incident infections (top rows in Figs. 3, A.1 and A.3) falls less steeply in subsequent birth cohorts (birth cohorts 2004–2029) as the vaccinated cohorts age and therefore join age groups with lower transmission rates. Fifteen years after the introduction of HepB3 vaccination (the 2015 birth cohort), women who were vaccinated in their infancy start becoming mothers, thereby providing indirect protection from vertical infection to newborns, which results in the percentage of direct vaccination effects for incident infections falling increasingly more steeply. The percentage of direct vaccination effects for incident infections increases sharply but transiently upon the introduction of HepB-BD vaccination (the 2030 birth cohort) in countries with the highest risks of vertical transmission (Moldova and Kosovo; Fig. 1(c)). This sharp jump in the percentage of direct effects in response to the initiation of HepB-BD is even more pronounced for incident chronic infections (Figure A.3) than for all incident infections (Fig. 3) because HepB-BD curtails vertical infections and a large proportion of vertical infections become chronic. Over time (the 2031 birth cohort onwards), the proportion of overall vaccination effects for incident infections that is direct (top rows in Figs. 3, A.1 and A.3) falls due to the increasing percentage of the population that is vaccinated and therefore immune and the concomitant fall in disease prevalence.

The countries with the highest percentage of direct vaccination effects in horizontal and vertical infections (Moldova and Kosovo; top rows in Fig. 3 and A.1) contain relatively small proportions of young children (Fig. 1(a)), contain women that tend to give birth at older ages (Fig. 1(b)), and seem to have the highest risks of vertical transmission (Fig. 1(c)). In these countries, young children constitute a relatively small proportion of the total population and so there will be very little protection from the disease due to vaccination present in the population, even after several years of vaccination (see the relatively slow decrease of HBsAg prevalence before the initiation of HepB-BD vaccination in Moldova and Kosovo relative to the other countries in the top row in Figure A.5). Hence, indirect protection from HepB3 vaccination plays a lesser role in preventing horizontal transmission in these countries compared to the other countries in the years after the initiation of the vaccination programme (Figs. 3, A.1, A.3 and A.6). A related effect makes the indirect effects of HepB3 vaccination lower in the same settings: where women tend to give birth at an older age, a greater proportion of mothers of newborns will be unvaccinated in the early decades of the vaccination programme. Hence, indirect protection from the HepB3 vaccination programme will play a more minor role in preventing vertical infections, resulting in a high percentage of direct protection in vertical infections after the introduction of HepB-BD vaccination (Figs. 3, A.1, A.3 and A.7). Direct vaccination effects therefore constitute a larger proportion of overall vaccination effects (between 2000 and 2070) in both vertical and horizontal infections in Moldova and Kosovo relative to the other countries (Figs. 3, A.1 and A.3). Since there is a high risk of vertical infection from infected women, the patterns in vertical infections (last rows in Figs. 3, A.1 and A.3) have a noticeable effect on the patterns in incident infections (horizontal and vertical combined; first rows in Figs. 3, A.1 and A.3) in these countries. The introduction of HepB-BD results in vaccination effects increasing suddenly in Moldova and Kosovo.

Because a HepB3 vaccination programme tends to involve immunity due to vaccination from horizontal infection being offered to the younger, more vulnerable age groups of a population, initiating a HepB3 vaccination programme will result in the younger age groups containing higher levels of immunity to horizontal infection, which in turn leads to the average age at which horizontal infection is acquired to rise over time. The average age of infection also increases due to the reduction in the FOI, which reduces each person’s chance of infection, thereby delaying the exposure of susceptible individuals (first row in Figure A.8). The average age of horizontal chronic infections (second row in Figure A.8) is lower than the average age for all horizontal infections (first row in Figure A.8), since the risk of a horizontal infection becoming chronic decreases steeply with age. Hence, the introduction of HepB3 and later HepB-BD causes the number of horizontal chronic infections to decrease relative to those horizontal infections that do not become chronic (Figure A.9).

**Fig. 4:**
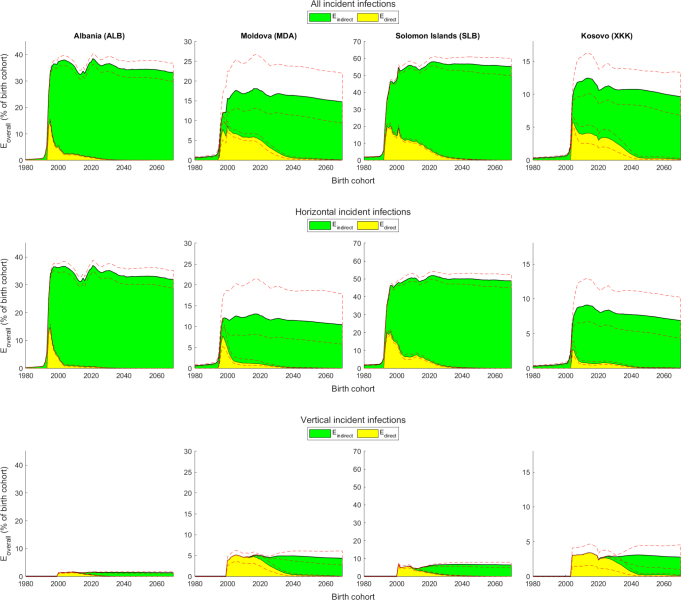
Vaccination effects ($E_{\text{overall}}$, $E_{\text{direct}}$ and $E_{\text{indirect}}$) amongst all infections (acute and chronic) under the aspirational vaccination scenarios. Vaccination effects (with 95% credible intervals indicated by red dashed lines) as a percentage of birth cohort size in all (top row), horizontal (middle row) and vertical (bottom row) incident infections under the aspirational vaccination scenarios.

### 3.2 Vaccination impact in the aspirational scenario

Including the vaccination scenarios involving aspirational vaccination coverage provides additional insights into how vaccination effects respond to vaccination coverage in the four countries. The percentage of direct vaccination effects for incident infections (Figs. 4, A.2 and A.4) falls steeply where HepB3 and HepB-BD are introduced in close succession at almost maximum coverage and vertical transmission is low (Albania; Fig. 1(c)). Administering HepB3 to infants provides indirect protection to young children in adjacent birth cohorts, thereby rapidly reducing the strength of direct protection in these vulnerable groups in subsequent birth cohorts. In contrast, vaccination coverage was scaled up more slowly in the Solomon Islands (see bottom row of Fig. 2), resulting in the percentage of direct effects for incident infections falling more slowly in Figure A.2. An initial high peak followed by a slower decline in the percentage of direct vaccination effects is seen in countries in which HepB3 and HepB-BD are introduced within close succession of each other and vertical transmission is high (Moldova and Kosovo), since neither HepB3 nor HepB-BD provide much indirect protection to babies born to infected mothers during the initial years of the vaccination programme. Over time, the proportion of overall vaccination effects for incident infections that is direct falls due to the increasing percentage of the population that is vaccinated and the concomitant fall in disease prevalence (Table 1). Overall vaccination effects are estimated to have prevented the following number of infections in the 1980 to 2030 birth cohorts due to HepB-BD and HepB3 vaccination in the countries: 522 thousand (494 thousand to 547 thousand) in Albania, 247 thousand (180 thousand to 355 thousand) in Moldova, 366 thousand (351 thousand to 386 thousand) in Soloman Islands and 57 thousand (46 thousand to 70 thousand) in Kosovo (Table 1). In 2030, the percentage of the overall effects attributable to direct vaccination effects is 0.8% (0.6% to 0.9%) in Albania, 13.3% (5.2% to 18.9%) in Moldova, 3.4% (2.6% to 4.1%) in Soloman Islands and 26.1% (12.5% to 34.6%) in Kosovo (top row in Figure A.2).

**Table 1:**
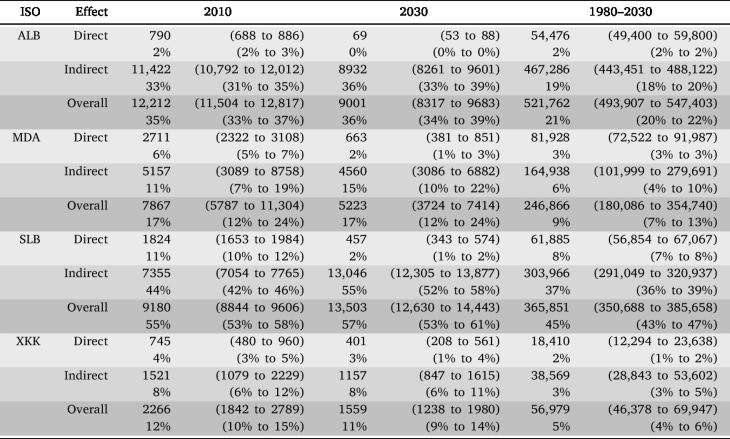
Incident infections (both vertical and horizontal) averted due to direct, indirect and overall vaccination effects under the aspirational vaccination scenarios in the 2010 and 2030 birth cohorts, as well as the cumulative effects between the 1980 and 2030 birth cohorts, with 95% credible intervals. The vaccination effect as a percentage of birth cohort size is also given. HepB-BD and HepB3 vaccination were each introduced in the four LMICs between 1993 and 2004 (see bottom row of Fig. 2). Hence, by 2010, both HepB-BD and HepB3 vaccination have recently been introduced in each country. ALB: Albania; MDA: Moldova; SLB: Soloman Islands; XKK: Kosovo.

## 4 Discussion

Vaccination is an effective intervention in the global struggle to eliminate hepatitis B. Not only does successful vaccination provide protection from infection to the vaccine recipient, but it also provides a level of protection to unvaccinated members of the community. However, it is evident from the current study that the level of indirect protection is dependent on the makeup of the population, with countries with large proportions of young children (which characterises most LMICs) displaying stronger indirect protection in the initial years of the vaccination programme (Albania and Soloman Islands versus Moldova and Kosovo). This is because countries with larger proportions of young children will have a greater proportion of their total population vaccinated than will countries in which older age groups dominate. We found that, irrespective of the demographic structure of the countries investigated, after the first 2–5 years, a sizeable proportion of overall protection was attributable to indirect protection. This finding highlights the importance of including indirect effects when modelling hepatitis B vaccination interventions, using dynamic models over static models which would only capture the direct effects and therefore underestimate impact (Wilder-Smith et al., 2017).

In previous work (de Villiers et al., 2021), we found HepB-BD to be very important in reducing hepatitis B burden, saving about 710,000 lives in the 2020 to 2030 birth cohorts in 110 LMICs if HepB-BD is scaled up to 90% by 2030 compared to keeping both HepB-BD and HepB3 vaccine coverage at status quo in each country. This current study identifies some population characteristics that make HepB-BD vaccination especially helpful in augmenting a well-established HepB3 vaccination programme. Including HepB-BD in the vaccination programme is important in populations with high levels of vertical transmission, since indirect protection from HepB3 takes about 25 years on average (see Fig. 1(b)) to reach newborns from their vaccinated mothers. This delayed benefit to newborns makes HepB-BD even more important in countries with higher fertility rates in older women. HepB-BD is also important in countries with relatively small numbers of young children. This is because vaccinated individuals make up a relatively small percentage of the total population during the initial years of the vaccination programme, resulting in relatively low indirect vaccination effects in these countries. There is therefore very little indirect protection from infection for women of childbearing age, and newborns are therefore at increased risk of vertical infection. In addition to factors such as HBsAg prevalence and the speed and final levels of vaccination scale-up, taking the aforementioned characteristics (risk of vertical transmission, fertility of older women, dominance of older age groups in the population) into account may explain why some of the countries in de Villiers et al. (2021) may take several decades to reach hepatitis B elimination if vaccination rates do not increase in the future.

This study also shows that not only does hepatitis B vaccination result in a drop in incident infections, it also results in the average age of infection increasing (Brisson and Edmunds, 2003). Since the risk of chronic infection falls with age (Edmunds et al., 1993), this leads to an even faster drop in chronic infections relative to those infections that do not become chronic. Hence, a population-wide hepatitis B vaccination programme results in a decline in the rate of infections and an even more rapid decline in the rate of chronic infections. This study also demonstrates the important role that HepB-BD plays in reducing chronic infections by targeting vertical infections.

The relative importance of indirect effects appears to grow at different rates depending on how long the vaccination programme has already been running, growing most rapidly while there are still unvaccinated cohorts amongst the age groups most responsible for the spread of horizontal infection (under-five-year-olds). As infection prevalence in the population decreases over time, the indirect vaccination effect becomes increasingly dominant relative to the direct vaccination effect. The increasing dominance of indirect effects confers greater protection to unvaccinated members of the population, which means that temporary interruptions to the vaccination programme (as was seen during the COVID-19 pandemic in many countries (Ghaznavi et al., 2023)) are less likely to result in immediate increases in incidence where programmes are mature. An important aspect of countries preparing for future pandemics is therefore to reduce the prevalence of prevalent vaccine-preventable communicable diseases to sufficiently low levels as soon as possible by scaling up vaccination coverage to very high levels.

### 4.1 Strengths and limitations of the study and future work

Mathematical modelling provides a good complement to the knowledge gained from clinical trials. Mathematical modelling makes it easier to partition the target population into vaccinated and unvaccinated groups in the case of the hepatitis B vaccine in which the timing and number of doses determines whether an individual is protected from vertical and/or horizontal infection. Moreover, infectiousness can last for several years and chronic hepatitis B infections tend to last for decades, lending themselves to mathematical modelling which handles long time spans easily. However, modelled populations are simplifications of real populations. By not taking population complexities into account, we assumed that results would average out across the population.

HepB3 coverage is high in most countries, and it is therefore important to understand the added impact of HepB-BD in hepatitis B prevention, which is the purpose of the stylised scenario. We do surmise the contribution of the constituent vaccines (HepB-BD and HepB3) in this study, but each contribution is in the presence of the other vaccine and should not be interpreted as the effect the vaccine would have in isolation.

Another characteristic of this study is that we modelled the vaccinated part of the target population as made up of successfully vaccinated individuals and individuals in which the vaccine failed. The former group was modelled as being completely protected from infection, while the latter group was modelled as being completely unprotected from infection i.e. as vulnerable to infection as unvaccinated individuals. An alternative approach would have been to model each interaction between a vaccinated and infectious person as having a pre-specified probability of resulting in an infection (Smith et al., 1984). Both of these approaches are over-simplifications; in reality, the protection conferred by vaccination depends on characteristics of the individual and probably ranges from the maximum efficacy of the vaccine to very low, and probably also wanes over time in some individuals.

Because the simulations were run to the year 2150, the birth cohorts born from 2050 onwards are missing their oldest age groups. However, since hepatitis B transmission rates are highest in young children, the premature truncation of birth cohorts towards the end of the simulations should result in only a slight downward bias in vaccination effects in the affected birth cohorts.

Future studies could explore the vaccination effect as well as how the ratio of direct to indirect vaccination effects changes in response to differing levels of each of HepB3 and HepB-BD vaccination coverages. A wider range of countries with a greater diversity of demographic and epidemiological characteristics could also be explored in order to identify more characteristics that affect vaccination effects. How these epidemiological dynamics help determine cost-effectiveness should also be investigated. However, given that our study included three countries from Europe, care must be taken about generalising our findings to countries in AFRO and WPRO, the WHO regions that contain the largest hepatitis B burdens (World Health Organization, 2024b), where there will be other demographic and epidemiological characteristics, including younger populations, higher fertility rates amongst younger women, higher burdens of disease, higher mortality rates amongst younger people, etc.

## 5 Conclusions

Hepatitis B is transmitted directly from person to person, and the hepatitis B vaccine therefore confers indirect protection to unvaccinated members of the population, with the proportion of direct and indirect effects depending on the characteristics of the population and the vaccination programme in complex ways. High HepB-BD coverage levels are especially impactful in populations that are more dominated by older age groups and/or have higher levels of vertical transmission — characteristics that suppress the role of indirect effects of HepB3 thereby making it more difficult for the benefits of HepB3 vaccination to reach all population groups during the first years of a HepB3 vaccination programme. HepB-BD is therefore a vital additional tool for reducing rates of vertical transmission quickly, thereby reaching elimination of hepatitis B globally more rapidly.

## CRediT authorship contribution statement

**Margaret J. de Villiers:** Writing – original draft, Visualization, Software, Methodology, Investigation, Formal analysis, Data curation. **Edward de Villiers:** Writing – original draft, Software, Methodology, Investigation, Formal analysis. **Shevanthi Nayagam:** Writing – review & editing, Supervision, Project administration, Funding acquisition. **Timothy B. Hallett:** Writing – review & editing, Supervision, Project administration, Methodology, Funding acquisition.

## Funding

This work was supported by the 10.13039/100000865Bill & Melinda Gates Foundation, via the Vaccine Impact Modelling Consortium [Grant Number INV-034281] . MJdV, SN, and TBH also acknowledge funding from the MRC Centre for Global Infectious Disease Analysis (reference MR/X020258/1), funded by the UK Medical Research Council (MRC). This UK funded award is carried out in the frame of the Global Health EDCTP3 Joint Undertaking. SN is also supported by the 10.13039/501100013342NIHR Imperial Biomedical Research Centre (BRC; https://imperialbrc.nihr.ac.uk/). EdV thanks the UK National Institute for Health and Care Research. The funder did not contribute to the design, analysis or interpretation of this study.

## Declaration of competing interest

This work was carried out as part of the Vaccine Impact Modelling Consortium (www.vaccineimpact.org), but the views expressed are those of the authors and not necessarily those of the Consortium or its funders. The funders were given the opportunity to review this paper prior to publication, but the final decision on the content of the publication was taken by the authors. SN reports grants to her institution not connected to this work from Gavi, the Vaccine Alliance. TBH reports grants to his institution not connected to this work from Gavi, the Vaccine Alliance. All other authors declare no competing interests.

## Data Availability

Data and code are accessible at: 10.5281/zenodo.13858436.

## Appendix A Supplementary data

MMC S1 The supplementary materials includes plots of vaccination effects, disease prevalence, and chronic to acute infections.
