# Supplementary material for: Direct and indirect effects of hepatitis B vaccination in four low- and middle-income countries
Source: Epidemics. 2024 Dec;49:100798. doi: 10.1016/j.epidem.2024.100798 (PMC11649532; doi:10.1016/j.epidem.2024.100798)
Supplement: MMC S1 — The supplementary materials includes plots of vaccination effects, disease prevalence, and chronic to acute infections. [file mmc1.pdf]

A Supplementary Material

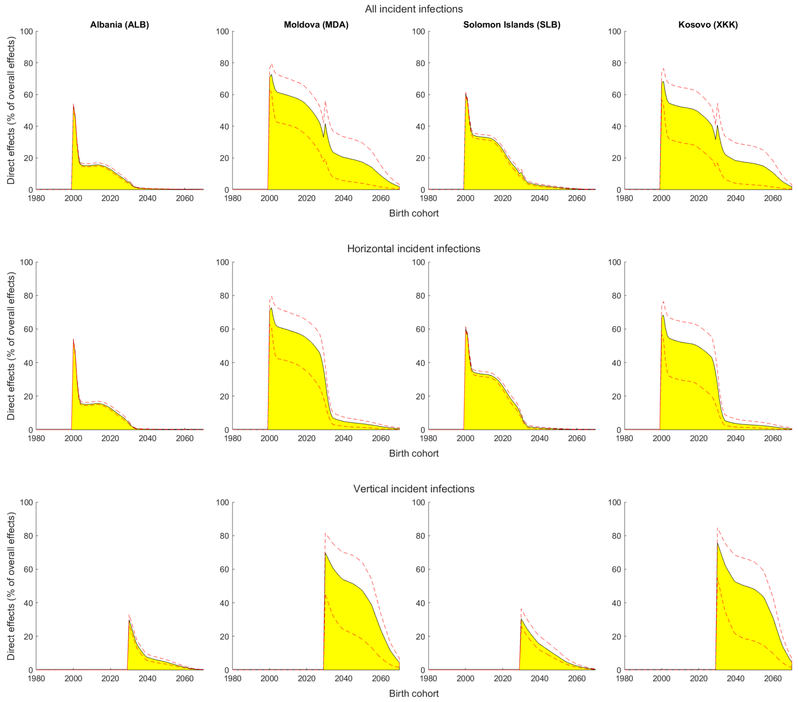

Figure A.1: Direct vaccination effects amongst all infections (acute and chronic) under the stylised vaccination scenarios. Direct vaccination effects (with 95% credible intervals indicated by red dashed lines) as a percentage of overall vaccination effects in all (top row), horizontal (middle row) and vertical (bottom row) incident infections under the stylised vaccination scenarios.

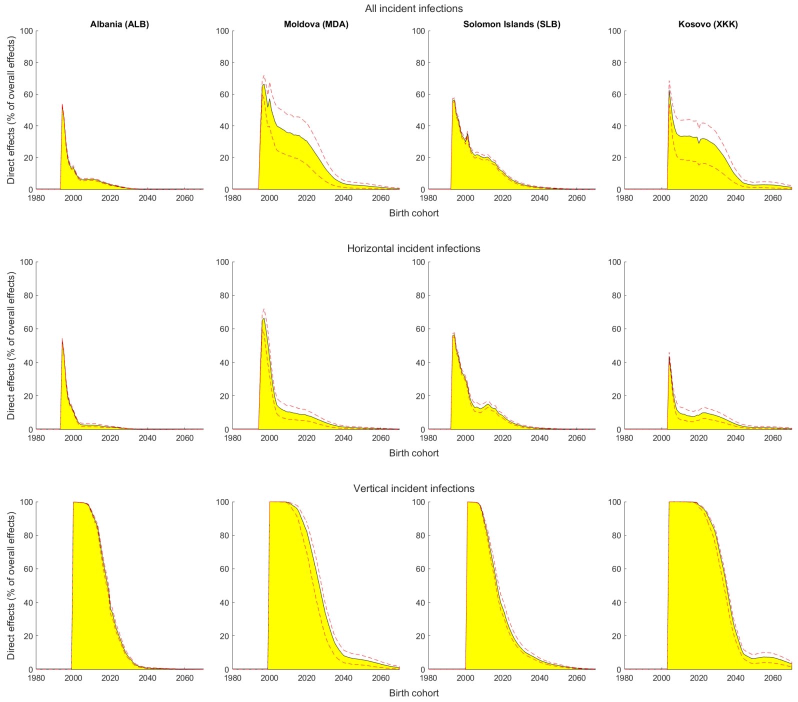

Figure A.2: Direct vaccination effects amongst all infections (acute and chronic) under the aspirational vaccination scenarios. Direct vaccination effects (with 95% credible intervals indicated by red dashed lines) as a percentage of overall vaccination effects in all (top row), horizontal (middle row) and vertical (bottom row) incident infections under the aspirational vaccination scenarios.

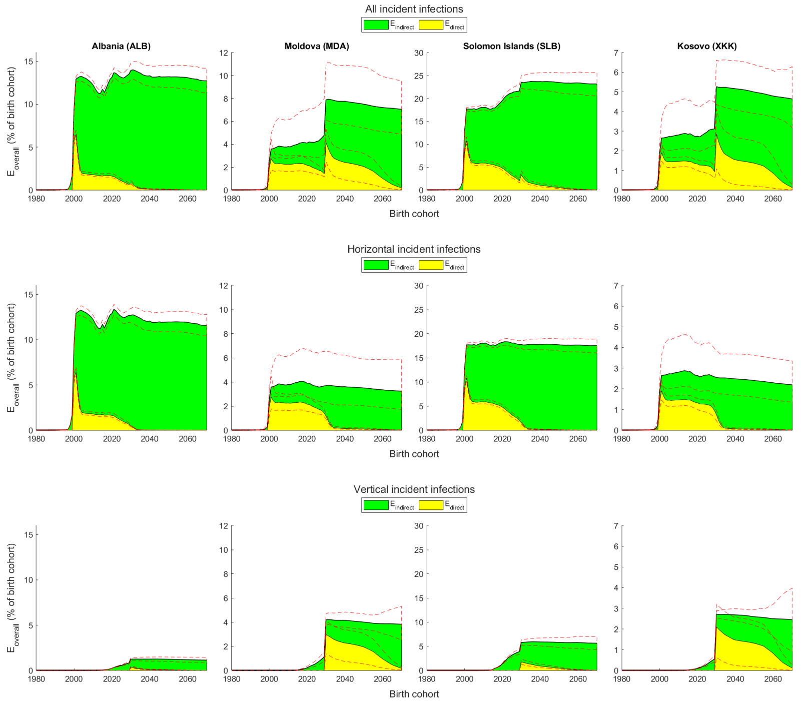

Figure A.3: Vaccination effects (E_{overall}, E_{direct} and E_{indirect}) amongst chronic infections under the stylised vaccination scenarios. Vaccination effects (with 95% credible intervals indicated by red dashed lines) as a percentage of birth cohort size in all (top row), horizontal (middle row) and vertical (bottom row) incident infections under the stylised vaccination scenarios.

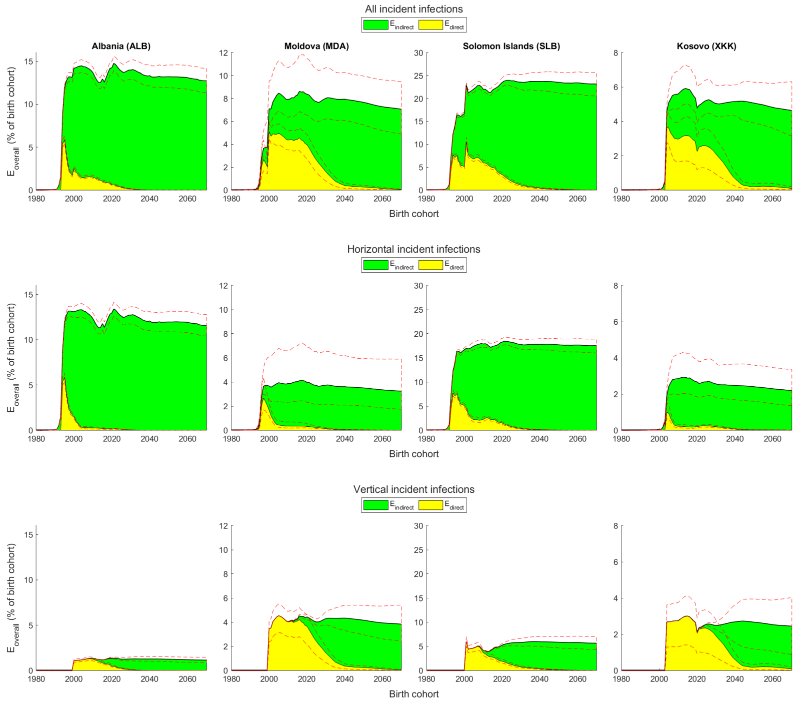

Figure A.4: Vaccination effects (E_{overall}, E_{direct} and E_{indirect}) amongst chronic infections under the aspirational vaccination scenarios. Vaccination effects (with 95% credible intervals indicated by red dashed lines) as a percentage of birth cohort size in all (top row), horizontal (middle row) and vertical (bottom row) incident infections under the aspirational vaccination scenarios.

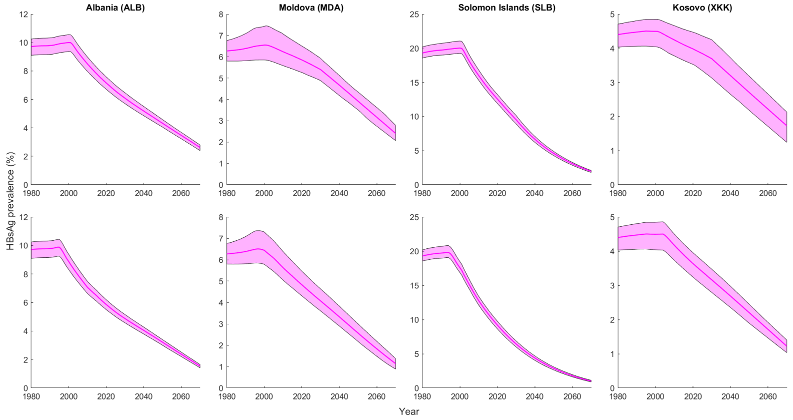

Figure A.5: HBsAg prevalence (with 95% credibility intervals) in the stylised (top row) and aspirational (bottom row) vaccination scenarios.

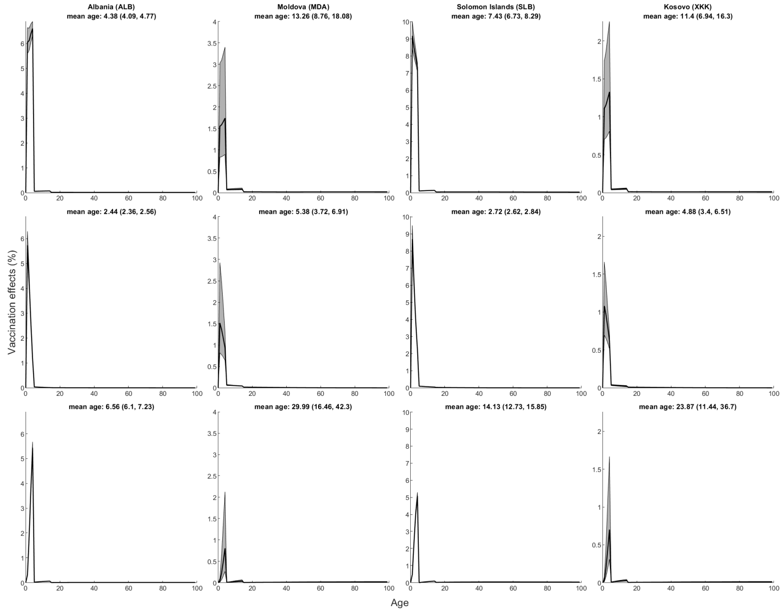

Figure A.6: Overall (top row), direct (second row), and indirect (last row) vaccination effects (with 95% credibility intervals) for all new infections (acute and chronic) in the 2000 birth cohort as a percentage of age group size under the stylised vaccination scenarios.

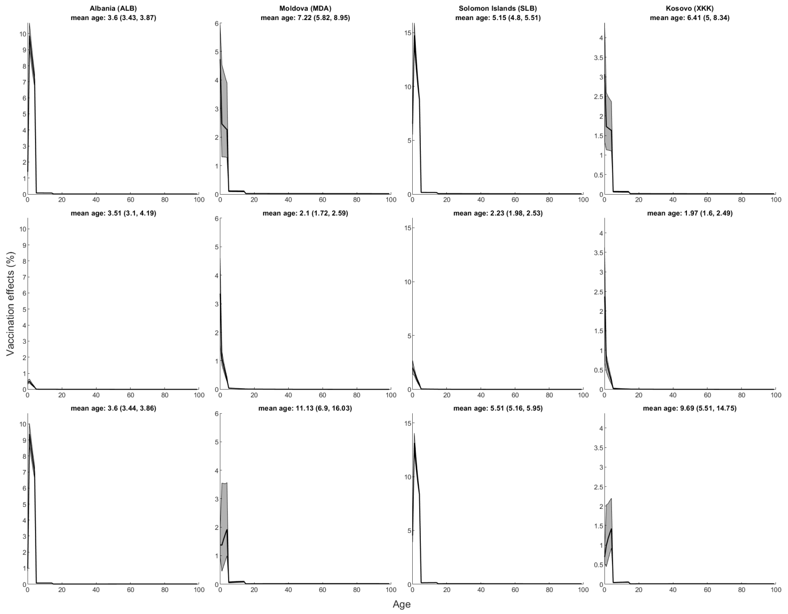

Figure A.7: Overall (top row), direct (second row), and indirect (last row) vaccination effects (with 95% credibility intervals) for all new infections (acute and chronic) in the 2030 birth cohort as a percentage of age group size under the stylised vaccination scenarios.

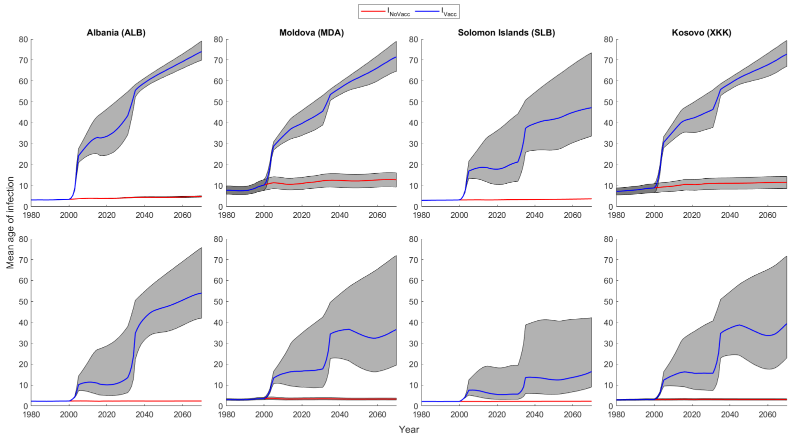

Figure A.8: Mean age (with 95% credibility intervals) of incident horizontal infections (top row) and incident horizontal chronic infections (bottom row) under the stylised vaccination scenarios.

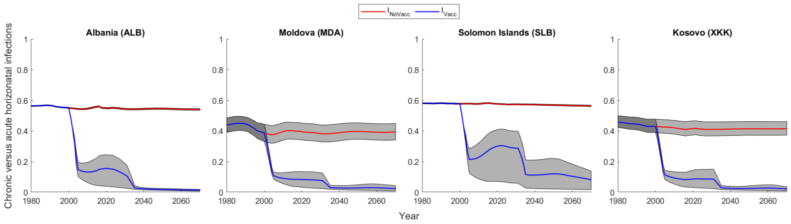

Figure A.9: Horizontal chronic infections relative to horizontal acute infections (with 95% credibility intervals) under the stylised vaccination scenarios.
